# Postpartum psychosis: A proposed treatment algorithm

**DOI:** 10.1177/02698811231181573

**Published:** 2023-07-29

**Authors:** Chaitra Jairaj, Gertrude Seneviratne, Veerle Bergink, Iris E Sommer, Paola Dazzan

**Affiliations:** 1 South London and Maudsley NHS Foundation Trust, London, UK; 2 Trinity College Dublin, Dublin, Ireland; 3 National Maternity Hospital, Dublin, Ireland; 4 Royal College of Psychiatrists, London, UK; 5 Department of Psychiatry, Icahn School of Medicine at Mount Sinai, New York, NY, USA; 6 Department of Obstetrics, Gynecology, and Reproductive Science, Icahn School of Medicine at Mount Sinai, New York, NY, USA; 7 Department of Psychiatry, Erasmus Medical Centre Rotterdam, Rotterdam, The Netherlands; 8 Department of Psychiatry, Rijksuniversiteit Groningen (RUG), University Medical Centre Groningen (UMCG), Groningen, The Netherlands; 9 Department of Psychological Medicine, Institute of Psychiatry, Psychology and Neuroscience, King’s College London, London, UK; 10 National Institute for Health Research (NIHR) Mental Health Biomedical Research Centre at South London and Maudsley NHS Foundation Trust and King’s College London, London, UK

**Keywords:** Postpartum psychosis, prophylaxis, treatment

## Abstract

**Background:**

Postpartum psychosis (PPP) is a psychiatric emergency that generally warrants acute inpatient care. PPP is marked by the sudden onset of affective and psychotic symptoms with a rapid deterioration in mental state. Evidence suggests that PPP is a discrete disorder on the bipolar disorder spectrum with a distinct treatment profile and prognosis.

**Methods:**

We conducted a PubMed database search for various terms involving PPP and its treatment and included peer-reviewed articles published in English.

**Objective:**

To provide a treatment algorithm for the management of PPP based on available evidence.

**Results:**

Pharmacological therapy is the mainstay of PPP management in the acute phase. Evidence points to a combination of antipsychotics and lithium in the acute treatment of PPP. Electroconvulsive therapy can offer a rapid treatment response where required. Lithium appears to have the best evidence for relapse prevention and prophylaxis in PPP. Psychoeducation is essential and psychosocial interventions used in bipolar disorder may be effective in PPP.

**Conclusion:**

Early detection and prompt treatment with antipsychotics and lithium, followed by maintenance treatment with lithium, is associated with a favourable prognosis in PPP.

## Introduction

Postpartum psychosis (PPP) is a severe mental disorder with a dramatic and rapid onset of affective and psychotic symptoms in the immediate postpartum period. PPP is a psychiatric emergency that generally warrants acute inpatient care (Wesseloo et al., 2015). Due to its severe and fluctuating nature, there is a significant risk to the mother and infant if left untreated, with potential for the tragic consequences of suicide or infanticide (Jones et al., 2014). With maternal suicide being the leading cause of direct maternal death occurring within the first postpartum year, early detection and management of postpartum mental illness is key to improving outcomes for the mother and her infant (Knight et al., 2020). Evidence indicates that PPP is a disorder on the bipolar spectrum in women with a vulnerability to the childbirth trigger (Perry et al., 2021). Although PPP is a discrete disorder on the affective disorder spectrum with a distinctive treatment profile, it is yet to be recognised as a separate nosological entity in the International Classification of Diseases, Tenth Revision and the Diagnostic and Statistical Manual of Mental Disorders, Fifth Edition (American Psychiatric Association, 2013; World Health Organisation, 2018). The primary aim of this review is to provide an evidence-based algorithm for the treatment of PPP, taking into account the unique management challenges during this vulnerable period for the mother–infant dyad.

## Method

A search was conducted in the PubMed database using the terms (‘postpartum psychosis’ [MeSH Terms] OR ‘postpartum psychosis’ [All Fields]). The terms (‘antipsychotics’ [MeSH Terms] OR ‘antipsychotics’ [All Fields]) AND (‘lactation’ [MeSH Terms] OR ‘lactation’ [All Fields] OR ‘breastfeeding’[MeSH Terms] OR ‘breastfeeding’ [All Fields]), and (‘lithium’ [MeSH Terms] OR ‘lithium’ [All Fields]) AND (‘lactation’ [MeSH Terms] OR ‘lactation’ [All Fields] OR ‘breastfeeding’ [MeSH Terms] OR ‘breastfeeding’ [All Fields]) were also searched. Peer-reviewed articles published in English were included in this review.

## Clinical presentation

PPP has a prevalence of 1–2 per 1000 births (Harlow et al., 2007; Kendell et al., 1987; Munk-Olsen et al., 2006; Valdimarsdóttir et al., 2009). A recent systematic review found the global prevalence of PPP to be 0.89–2.6 per 1000 births, although the data largely represented women from high-income countries (VanderKruik et al., 2017). Another systematic review examined data from low- and middle-income countries and reported PPP prevalence rates of 1.1–3.2 per 1000 births, indicating a similar prevalence globally (Kalra et al., 2022).

The onset of PPP is usually rapid (within hours), with most episodes beginning between days 3 and 10 postpartum (Bergink et al., 2011a; Klompenhouwer and Hulst, 1991). Early warning symptoms can include insomnia, anxiety, irritability or mood fluctuation, with elated or depressed mood, confusion, perplexity and disorganised behaviour emerging later. Persecutory delusions and delusions of reference are common. Visual hallucinations, disorientation and delirium-like symptoms are reported to occur more frequently in PPP than in non-postpartum psychotic disorders (Ganjekar et al., 2013; Gordon-Smith et al., 2020; Kamperman et al., 2017). Delusions and hallucinations can involve the infant and may sometimes be concealed due to a lack of insight or the presence of stigma (Chandra et al., 2006).

Catatonia is common in PPP with prevalence rates ranging from 5% in a Dutch study of 130 women with PPP to 20% in an Indian study of 200 women with PPP (Kamperman et al., 2017; Nahar et al., 2017). Mutism, withdrawal and negativism are the most prevalent features of catatonia in PPP (Nahar et al., 2017). A longer duration of untreated catatonia in PPP has been associated with poor response to lorazepam in one study, highlighting the importance of early detection and treatment during this period (Nahar et al., 2017).

Mental state can fluctuate in PPP, and insight is generally poor. High vigilance, robust social support and close monitoring are paramount in this period, particularly in women with risk factors for developing PPP. Given the rapidly changing mental state and risk to the mother and the infant, admission is indicated, ideally to a mother and baby unit (MBU) where available (Bergink et al., 2016; Jones et al., 2014).

## Potential consequences of maternal PPP on the infant

PPP can have negative consequences for both mother and infant. Delusional ideation often relates to the infant and may lead to (often risky) maternal protective behaviours or abuse/neglect of the infant (Chandra et al., 2006). In an Indian study, 43% of women with PPP reported infanticidal thoughts and 36% reported infanticidal behaviour (Chandra et al., 2002). In contrast, a Dutch study reported infanticidal thoughts in 8% of women with PPP (Kamperman et al., 2017), highlighting possible sociocultural differences in risk or expression of risk to infants in PPP. Sociodemographic factors including maternal early life adversity, socioeconomic disadvantage, domestic violence and substance misuse are associated with infanticide (Friedman et al., 2005).

PPP can also negatively affect mother–infant bonding, an interaction that begins in pregnancy, and is important for future infant attachment and maternal role gratification (Sharmila et al., 2019). One study examined maternal perceived bonding in women at risk of PPP and found that women at risk who developed PPP had a negative affective experience towards their pregnancies antenatally and towards their infants postnatally, compared to women at risk who remained well postpartum (Biaggi et al., 2021). Another recent study found impaired self-reported mother–infant bonding in 17.6% of women with PPP admitted to an MBU and reported a strong association between a reduction in psychiatric symptoms and improved bonding (Gilden et al., 2020b). A small group of women with PPP (0.05%) in that study continued to experience impaired bonding even after remission of psychiatric symptoms.

## Risk factors for PPP

Due to its heterogeneity and relatively low prevalence, the aetiology of PPP remains poorly understood. A detailed discussion of the aetiology of PPP is out of the scope of this review but has been discussed elsewhere (see Perry et al., 2021).

The strongest risk factors for developing PPP are a history of bipolar disorder and/or past episodes of PPP (Di Florio et al., 2018; Jones and Craddock, 2001; Munk-Olsen et al., 2009; Viguera et al., 2011). It is becoming increasingly evident that PPP lies on the same spectrum as bipolar disorder. Rates of PPP in women with bipolar disorder vary widely. They have been reported to range from 8.5% in a population-based Swedish registry study to 75% in another study of women who were medication-free during the perinatal period (Di Florio et al., 2013; Doyle et al., 2012; Harlow et al., 2007; Maina et al., 2014). Women with bipolar I disorder have a higher risk for PPP compared to women with bipolar II disorder (Di Florio et al., 2013). There is no literature on relapse risk in women with a history of both bipolar disorder and a history of PPP, but the risk is speculated to be highest in this group.

In addition to a personal history of bipolar disorder or PPP, having a first-degree relative with bipolar disorder or a history of PPP also confers an increased risk of developing PPP (Jones and Craddock, 2001). A recent study of polygenic risk scores in 203 women with first-onset PPP found that, compared to controls, genetic vulnerability for bipolar disorder was similar in women with PPP and those with bipolar disorder (Di Florio et al., 2021). However, examination of polygenic risk scores for major depression showed that women with PPP had significantly lower genetic vulnerability for major depression than those with bipolar disorder. These results further contribute to the growing evidence base for PPP being a separate nosological entity on the bipolar disorder spectrum.

Childbirth is a robust biological precipitant for PPP. Sleep deprivation during labour and postpartum is also a risk factor for the onset of PPP, particularly in women with a history of mania induced by sleep loss (Lewis et al., 2018). The only consistent obstetric risk factor for the development of PPP is primiparity (Di Florio et al., 2014). There is inconsistent evidence for maternal age as a risk factor for developing PPP. A large-scale study reported higher risk in younger women while another large study reported higher risk in maternal age over 35 (Swift et al., 2020; Valdimarsdóttir et al., 2009). Another study found no association between maternal age and PPP (Di Florio et al., 2014).

Non-psychiatric postpartum complications such as infection and lactation problems are risk factors for PPP and other postpartum psychiatric disorders (Hellerstedt et al., 2013). While a previous study found no link between psychosocial risk factors such as stressful life events and PPP (Dowlatshahi and Paykel, 1990), a more recent study found that severe childhood maltreatment and higher daily cortisol (a marker of stress response) in the third trimester of pregnancy predicted relapse in the first 4 weeks postpartum in women at risk of PPP (Hazelgrove et al., 2021).

## Differential diagnoses

The early presentation of PPP can be similar to that of other psychiatric disorders occurring in the puerperium. Postpartum blues can initially have a similar presentation to PPP with mood swings, insomnia and anxiety, but these symptoms usually self-resolve within a few days. Symptoms of PPP tend to worsen over time, usually rapidly. Postpartum depression may also have initial features in common with PPP. Early postpartum onset may be seen in postpartum depression, although it is not typical, which may differentiate from PPP (Stowe et al., 2005).

Medical causes for psychosis in the postpartum period should be ruled out following an assessment of the patient’s history, physical examination and investigations, including full blood count, metabolic profile, thyroid profile, urine drug screen, serum calcium levels, B12, folate and thiamine (Raza and Raza, 2021; Sit et al., 2006). Early symptoms of fluctuating confusion can be similar to symptoms of delirium. Infections such as mastitis or endometritis, delirium induced by eclampsia, metabolic or electrolyte abnormalities, and postpartum thyroiditis should be considered among the differential diagnoses (Bergink et al., 2016). Sheehan syndrome, a disorder of hypopituitarism resulting from postpartum haemorrhage, can lead to cognitive slowing and should be ruled out (Diri et al., 2016).

Immune system-mediated factors have been implicated in the onset of PPP (Bergink et al., 2013; Dazzan et al., 2018). In one study, 19% of women with first-onset PPP had concurrent autoimmune thyroid dysfunction (Bergink et al., 2011b). If suspected, autoimmune thyroid dysfunction should be ruled out with the assessment of anti-thyroid peroxidase antibodies. Autoimmune encephalitis was reported in 4% of women with PPP, 2% of which had anti-N-methyl-D-aspartate (NMDA) receptor encephalitis (Bergink et al., 2015a). Autoimmune encephalitis should be ruled out with the evaluation of serum anti-NMDA antibodies and neuroimaging, especially in the presence of concurrent neurological symptoms (Bergink et al., 2015a). A recent study also showed that women with PPP were more likely to have elevated C-reactive protein, an overall marker of systemic inflammation, compared to healthy postpartum women (Aas et al., 2020).

Medication or other drug-induced psychosis should also be considered in the organic aetiology of PPP and ruled out with a urine drug screen in all women affected (Jones et al., 2014). Evidence on brain structural and functional correlates of PPP remains scant (Fusté et al., 2017; Kowalczyk et al., 2021; Sambataro et al., 2021), and there is no evidence to date to support the use of neuroimaging as a routine investigation in this disorder.

## Prevention and prophylaxis

### Screening and prevention

Women have a 23 times higher risk for experiencing a first episode of an affective psychosis in the postpartum period than at any other period (Munk-Olsen et al., 2006). Early identification of the signs of PPP in the postpartum period can reduce the duration of the psychotic illness and improve outcomes by alleviating the impact of PPP on the mother–infant dyad. There are currently no screening tools specific to PPP, but the use of the Mood Disorder Questionnaire (MDQ), a brief self-report screening tool used to detect bipolar disorder, has been advocated (Clark et al., 2015). The MDQ takes approximately 5 min to complete and incorporates 13 symptoms, as well as information on the timing of symptoms and the degree of impairment. The Spanish version of the 16-item Prodromal Questionnaire was found to have good construct validity as a screening tool for PPP in a Peruvian population of pregnant women (Levey et al., 2018). However, screening tools are not routinely used in clinical practice, where the diagnosis is reliant on collateral history and mental state examination.

In women at risk of developing PPP, relapse prevention and management are the most desirable strategies to improve outcomes for women and their infants. Personal or family history of bipolar disorder or PPP are strong risk factors for developing PPP. Identifying women at risk of PPP, offering preconception counselling and ensuring appropriate management and support during pregnancy can reduce the risks and impact of PPP. Preconception counselling is essential in women with established bipolar disorder or a history of PPP, as this initial psychiatric assessment will inform management and treatment decisions during the perinatal period. Most important in preventing relapse is the provision of pharmacological prophylaxis in the immediate postpartum period.

Pregnant women at risk of developing PPP should ideally be referred to a specialist perinatal mental health service where available. Developing an integrated perinatal mental healthcare plan with input from the patient, her family support and other professionals involved in her care including maternity services, community mental health teams, and, if involved, social care services, is important for the best outcome for the mother–infant dyad. The perinatal mental healthcare plan will include plans for pregnancy, delivery and the immediate postpartum period.

### Prophylaxis during pregnancy

Different strategies for prophylaxis may be necessary for women with a history of bipolar disorder and those with a history of PPP (Bergink et al., 2016). While women with isolated episodes of PPP are at increased risk for subsequent postpartum episodes, one study found no risk of episodes *during* pregnancy, even when women do not take medications during pregnancy (Bergink et al., 2012). Thus, there is no indication for women with isolated episodes of PPP to start prophylactic medication during pregnancy, although they should be monitored during this period.

A detailed discussion of the management of bipolar disorder in the perinatal period is beyond the scope of this review. We recommend referring to evidence-based guidelines such as the British Association of Psychopharmacology (BAP) guidelines on the management of perinatal mental illness and National Institute for Health and Care Excellence (NICE) guidelines on antenatal and postnatal mental health (McAllister-Williams et al., 2017; National Institute for Health and Care Excellence, 2014a). Updated information on foetal and infant effects of individual psychotropic drugs in pregnancy can be found in databases such as the UK Teratology Information Service (http://www.uktis.org/html/exposures_-%20stu.html) and the List of Pregnancy Exposure Registries (https://www.fda.gov/science-research/womens-health-research/list-pregnancy-exposure-registries).

### Prophylaxis in the postpartum period

In women with a history of PPP, prophylactic antipsychotic medications or lithium should be started immediately after delivery to reduce the risk of relapse (Bergink et al., 2012, 2016). There is an argument for introducing prophylaxis 2–4 weeks before delivery as a preventative measure in some cases of PPP, as postpartum prophylaxis is thought to come too late. However, there is no available data at present to support this practice. Lithium has the most robust evidence base for relapse prevention in PPP and may be considered the medication of choice for prophylaxis (Bergink et al., 2012). Physiological changes in the perinatal period affect lithium pharmacokinetics and necessitate more frequent lithium blood level monitoring and dose adjustments in the perinatal period (Wesseloo et al., 2017). Lithium levels should be checked twice weekly in the first 2 weeks postpartum, and target lithium levels should be high (0.8–1.0 mmol/L) during the first month postpartum for relapse prevention (Poels et al., 2018). A cohort study suggests that rates of relapse are low up to 9 months postpartum in women with PPP treated with lithium (Burgerhout et al., 2017). Lithium dose may start to be tapered after 3 months postpartum but should be continued for 1 year postpartum for sustained remission (Payne, 2019; Poels et al., 2018).

There is evidence from a small sample study for the efficacy of olanzapine in the prophylaxis of PPP in the early postpartum period, although this needs replication in larger studies (Sharma et al., 2006). The sedative side effects of olanzapine may be beneficial in improving sleep. Psychoeducation about minimising the risk of recurrence, with information about sleep hygiene and the use of medication to help reduce sleep loss around the time of delivery and in the early postpartum period is also important.

Breastfeeding is an important consideration in women at risk of developing PPP.

While the risks and benefits of prophylactic medication and the woman’s preference for breastfeeding should be considered, it is important to bear in mind that sleep deprivation associated with breastfeeding can precipitate a relapse of PPP. Lithium crosses into breast milk in large quantities and can have an effect on the infant (McAllister-Williams et al., 2017). Low levels of olanzapine are found in breast milk, and undetectable levels have been reported in the serum of breastfed infants (LactMed, 2022). Infant sedation has been reported with olanzapine (McAllister-Williams et al., 2017). Therefore, women with a history of PPP should be advised that breastfeeding, if it prevents adequate pharmacological prophylaxis and affects sleep, may increase the risk of relapse, with subsequent negative effects on the mother–infant dyad. Please see the section on breastfeeding below for a detailed discussion.

### Management

**Figure 1.**
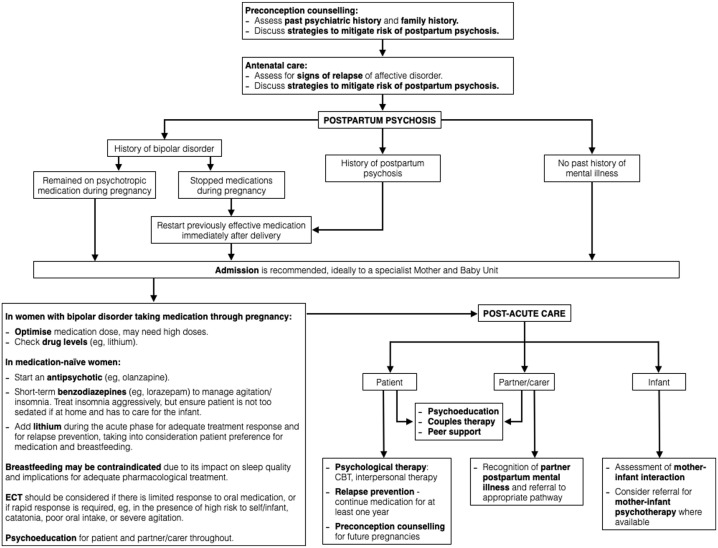
Treatment algorithm for PPP. CBT: cognitive behavioural therapy; ECT: electroconvulsive therapy; PPP: postpartum psychosis.

Given the acuity and risk involved, PPP is a psychiatric emergency. Pharmacological treatment should be initiated immediately, and admission should be sought, preferably to an MBU where available (Figure 1). The recommendations for treatment of PPP should be personalised to each woman and her circumstances, taking into consideration the level of support a partner/carer may be able to provide overnight and the presence of other children needing care, among other family and social factors. Involvement of the patient’s partner/carer or other family members is helpful in care planning. The immediate goals of treatment are to alleviate psychiatric symptoms and manage risk. Longer-term goals involve supporting the mother in improving self-esteem, confidence and maternal role gratification, in addition to supporting infant health and development by promoting a positive mother–infant relationship. Offering preconception counselling before planning future pregnancies may significantly reduce morbidity for a bipolar course of illness (Heron et al., 2012).

### Assessment

A full psychiatric history and collateral history from the partner, maternity ward staff or carers should be obtained. Risk of suicide and infanticide should be ascertained, bearing in mind that risk is rapidly dynamic in the postpartum period. Enquiring about the patient’s thoughts and feelings towards her infant, how she feels she is bonding with her infant and any thoughts about protecting herself, her infant or the world around her can inform risk assessment. Physical examination and investigations will rule out differential diagnoses as described above.

### Inpatient admission

Admission to an inpatient setting is recommended for women with PPP. The mother and infant should not be left alone due to the rapidly changing and unpredictable nature of risk in PPP, with a kaleidoscopic picture of fluctuating mood and psychotic symptoms (Luykx et al., 2019). MBU admissions, where available, are associated with better clinical outcomes, reduced time to recovery and improvement in maternal ability to care for the infant (Buist et al., 2004; Glangeaud-Freudenthal et al., 2011; Meltzer-Brody et al., 2013; Stephenson et al., 2018). Where an MBU is not available, admission to an inpatient mental health unit is recommended, although this will be disruptive to mother–infant bonding. In such cases, depending on a dynamic risk assessment, the patient may be provided supervised access to her infant. These meetings should take place in an appropriate room outside the mental health unit. In less severe cases of PPP where two trusted adults are available to care for the mother and infant, a decision may be made to treat the woman in the community, with daily multidisciplinary team reviews and support in the acute phase.

### Breastfeeding

The risks and benefits to the mother–infant dyad have to be considered when breastfeeding recommendations are made in PPP. Although breastfeeding is recommended where possible, we argue that in PPP, the risks of breastfeeding to the mother–infant dyad may often outweigh the benefits. Sleep deprivation is common in breastfeeding women and can lead to deterioration of symptoms in PPP (Sharma, 2003). The choice of medications that may be used to treat PPP is limited in breastfeeding (e.g. lithium, with the best evidence base for treatment of PPP, is a relative contraindication to breastfeeding in many healthcare systems). Please see section ‘Lithium and other mood stabilisers’ for further discussion of lithium and breastfeeding), with additional constraints on doses of medications that may be used. The resulting inadequate treatment of PPP combined with the perpetuating effect of sleep deprivation can prolong the episode of PPP, further impacting the mother–infant dyad.

We acknowledge that in certain situations, such as low- and middle-income countries, formula feeding may not be feasible due to the costs of infant formula and hygiene concerns with bottles (Galbally et al., 2018). Breast milk is vital for the development of preterm infants and the use of a breast milk bank where available might be considered in this circumstance. Occasionally, when the prescribed medication is safe in breastfeeding, daytime breastfeeding may be supplemented by nocturnal formula feeds offered by the partner or carer to facilitate maternal sleep. Reference to the latest guidance on medications in breastfeeding is recommended before prescribing. There is limited long-term safety data for psychotropic medication in breastfeeding. Information on the levels of individual drugs in breast milk and infant blood and the possible adverse effects in the breastfeeding infant may be found, in databases such as the Drugs and Lactation Database (LactMed) [https://toxnet.nlm.nih.gov/newtoxnet/lact-med.htm].

### Pharmacological treatment in the acute phase

Pharmacological management is the mainstay of acute treatment for PPP (Figure 1). However, there is limited evidence available for the safety of medications in the perinatal period. Concerns about foetal and neonatal safety have led to the exclusion of pregnant and breastfeeding women from clinical drug trials since the thalidomide tragedy in the 1960s. During the perinatal period, women are often required to consider the ethical implications of medication use without robust safety data. Recent ethical guidelines for clinical trials advocate for the inclusion of pregnant and breastfeeding women in clinical trials, promoting studies designed to obtain knowledge relevant to the health needs of these women, undertaken after careful consideration of the best available data (Council for International Organisations of Medical Sciences, 2016; ICH Harmonised Guideline, 2019). With these recent changes, there is hope for more robust safety and efficacy data in the future for medications in the perinatal period. Current recommendations, however, are based on prospective clinical cohorts, retrospective studies, surveillance data and case reports.

Helpful resources on the use of psychotropic medication postpartum include the BAP consensus guidance on the use of psychotropic medication in pregnancy and postpartum (McAllister-Williams et al., 2017) and the NICE guideline on antenatal and postnatal mental health (National Institute for Health and Care Excellence, 2014a). Decisions regarding medication use in the perinatal period must be made following a full individual risk/benefit analysis, including an evaluation of the risks and benefits of accepting treatment and the risks of untreated mental illness to the mother–infant dyad.

### Benzodiazepines and hypnotics

As insomnia can lead to deterioration of symptoms in PPP, adequate sleep is of primary importance (Sharma, 2003). Hypnotics and benzodiazepines may be used to treat insomnia and agitation. Low doses of benzodiazepines with a short half-life and no active metabolites are recommended in breastfeeding (Kronenfeld et al., 2017). Lorazepam is not thought to cause any adverse effects in breastfeeding infants and is the preferred benzodiazepine in breastfeeding mothers (LactMed, 2022). Promethazine is often used in clinical practice, but there are no studies examining promethazine in PPP. Promethazine can lower basal prolactin secretion and may interfere with the establishment of lactation in the early postpartum period (LactMed, 2022).

There is limited data on zolpidem safety in breastfeeding, but with low levels in breast milk and a short half-life, infant adverse effects are thought to be unlikely (Fortinguerra et al., 2009). There is no safety data on zopiclone in breastfeeding. Breastfed infants should be monitored for sedation, hypotonia and respiratory depression, particularly with regular use of large doses of hypnotics (LactMed, 2022).

### Antipsychotics

Antipsychotics are beneficial in the acute treatment of PPP. Second-generation antipsychotics, with the exception of clozapine, are relatively safe in breastfeeding (Uguz, 2016). Olanzapine has the largest evidence base among antipsychotics in breastfeeding. Most of the evidence comes from a global safety surveillance report on olanzapine in breastfeeding by the pharmaceutical company Eli Lilly (Brunner et al., 2013). Adverse events were reported in 15.6% of infants exposed to olanzapine through breast milk, with somnolence (4%), irritability (2%) and tremor (2%) being most commonly reported (Brunner et al., 2013). Other smaller studies have reported a low relative infant dose for olanzapine and an absence of adverse effects in infants (Croke et al., 2002; Gardiner et al., 2003; Gilad et al., 2011). With a low relative infant dose, quetiapine is also considered safe in breastfeeding (LactMed, 2022). There are limited data on risperidone, and with a higher relative infant dose than olanzapine and quetiapine, use in breastfeeding should be considered with caution (LactMed, 2022). Limited long-term follow-up data suggest normal development in infants exposed to olanzapine and quetiapine (LactMed, 2022).

While antipsychotics are useful in the acute treatment of PPP and often preferred in clinical practice, only 50% of patients taking antipsychotic monotherapy have been found to remain in remission 9 months after the initial PPP episode (Bergink et al., 2015b). Antipsychotics used in conjunction with lithium have been reported to result in better outcomes (Bergink et al., 2016).

### Lithium and other mood stabilisers

There is evidence for lithium as an adjunctive medication in PPP for both acute treatment and prophylaxis, and a case report suggests it may also be effective in monotherapy in acute PPP (Bergink et al., 2016; Lichtenberg et al., 1988). In a study of 64 women with PPP, 98.4% achieved clinical remission with a combination of benzodiazepine, antipsychotic and lithium (Bergink et al., 2015b). In this stepwise treatment study, 6.3% achieved remission with a benzodiazepine alone, 18.8% achieved remission with a combination of benzodiazepine and antipsychotic, and 73.4% achieved remission with a combination of benzodiazepine, antipsychotic and lithium. The final step of the study algorithm was ECT, which was not used as all participants had responded to the combination of benzodiazepine, antipsychotic and lithium. In the same cohort, 79.7% of women maintained remission at 9 months postpartum with lithium monotherapy (Bergink et al., 2015b).

Lithium has the most robust evidence base for relapse prevention in PPP (Bergink et al., 2012). There are concerns about declining rates of lithium prescription worldwide at present, with preference given to second-generation antipsychotics in the treatment of bipolar disorder (Bauer, 2022). Similarly, in PPP, antipsychotics are routinely preferred in clinical practice for long-term relapse prevention, possibly because of clinicians’ negative perception of lithium’s side effect profile and requirement for blood monitoring. However, given the metabolic risks of antipsychotics in women with PPP and the superiority of evidence for lithium in relapse prevention, we recommend consideration of lithium for relapse prevention in PPP.

There are no studies examining the mood stabilisers lamotrigine or carbamazepine in the treatment of PPP. Sodium valproate should not be used in women of reproductive age (Sisodiya, 2018). Valproate use in pregnancy carries a significant risk of congenital malformations (Bromley et al., 2013; Meador et al., 2008; Weston et al., 2016; Wyszynski et al., 2005), developmental disability and reduction in IQ (Bromley et al., 2014; Meador et al., 2009, 2013), and increased risk of autism spectrum disorders and attention-deficit/hyperactivity disorder in infants (Christensen et al., 2013; Cohen et al., 2013).

### Lithium and breastfeeding

Lithium is excreted in varying quantities into breastmilk (McAllister-Williams et al., 2017) and is considered a contraindication to breastfeeding, although there is some controversy in this regard. Some evidence suggests that lithium may not be an absolute contraindication in breastfeeding, particularly in lithium monotherapy in infants over 2 months of age (Hermann et al., 2019; LactMed, 2022; Newmark et al., 2019; Uguz and Sharma, 2016). Infant serum lithium concentrations were compared among different feeding strategies (exclusive breastfeeding, formula feeding, and combination breast and formula feeding; *n* = 8 participants in each group) in a recent study of women with bipolar disorder taking lithium and their infants (Imaz et al., 2021). There were no adverse infant effects reported in this study in the first 2 months postpartum. No sustained lithium accumulation was reported in the three groups and infant lithium clearance was independent of maternal lithium levels. Infant lithium levels were reported to decline in all groups, although levels in exclusively breastfed infants took up to 60 days to fall below the limit of quantification, while formula-fed and combination-fed infants reached this level by 6–8 days postpartum. The authors recommend checking infant serum lithium levels at 2 days and 1 week postpartum, with further checks at 1 and 2 months postpartum in breastfeeding infants. While combination of breast and formula feeding may be considered relatively safe from the results of this study, further evidence from larger cohorts are necessary before it can be recommended.

If lithium is prescribed during breastfeeding, this should be done in conjunction with a neonatologist/paediatrician. While there are no reports of lithium toxicity or developmental problems in infants, infant serum lithium levels, serum creatinine, blood urea nitrogen levels and thyroid function should be monitored regularly in breastfed infants exposed to lithium as lithium can affect renal and thyroid function in infants (LactMed, 2022). Lithium may adversely affect breastfed infants when elimination is impaired, particularly in neonates and premature infants.

To summarise, PPP often requires polypharmacy and commonly occurs within the first 2 weeks postpartum, a period for which there is limited evidence for the safe use of lithium in breastfeeding. In the absence of more robust evidence on risks to the infant, our recommendation follows that of Galbally et al. (2018), suggesting that women prescribed lithium should abstain from breastfeeding.

### Antidepressants

There are no studies examining the efficacy of antidepressants in episodes of PPP characterised by severe depression with psychotic features. Some women with PPP may experience depression in the post-acute phase (Gilden et al., 2020a). Antidepressants are routinely prescribed, although there are no studies examining the use of antidepressants in PPP. Depression following an episode of PPP should be treated as bipolar depression. There is evidence for benefit of quetiapine, lamotrigine, lurasidone and olanzapine in bipolar depression (Goodwin et al., 2016). If the woman is already on lithium for PPP, lithium levels should be checked. If antidepressants are considered in women with a history of PPP, they should be prescribed along with a drug for mania, and should be tapered and discontinued when full remission of depressive symptoms has been achieved (Goodwin et al., 2016).

### Electroconvulsive therapy

Extant data suggest that the rapid treatment response following electroconvulsive therapy (ECT) may be beneficial in PPP (Babu et al., 2013; Doucet et al., 2010). The response rate for ECT is reported to be higher in the postpartum period than outside this period (Reed et al., 1999; Rundgren et al., 2018). One study found lower rates of hospitalisation and suicidality in women with postpartum depression and PPP receiving ECT compared to women receiving ECT outside the perinatal period (Rönnqvist et al., 2019). A naturalistic study of 78 patients with PPP showed that 43.6% of these patients received ECT with good response (Babu et al., 2013). Indications for ECT in that study included augmentation to pharmacotherapy in treatment-resistant PPP (defined as minimal response to pharmacotherapy after 2 weeks), catatonia, and suicidal or infanticidal behaviour. In a case series of five women with postpartum affective disorders, two women had treatment-refractory PPP and showed rapid improvement in psychotic symptoms after three sessions of ECT (Forray and Ostroff, 2007).

Transient anterograde amnesia has been reported as an adverse effect of ECT in women with PPP (Babu et al., 2013; Forray and Ostroff, 2007). One study reported prolonged seizures which responded to treatment with barbiturates (Babu et al., 2013). Women receiving ECT can continue to breastfeed their infants with no adverse effects on the infant (Babu et al., 2013; Forray and Ostroff, 2007).

Although ECT is considered an option of last resort for treatment-resistant PPP in many healthcare systems, we recommend earlier consideration of ECT in women with severe symptoms, suicidal/infanticidal ideation, catatonia or severe agitation. ECT can be useful in these cases where rapid treatment response is required, and may lead to better outcomes for the mother–infant dyad (Focht and Kellner, 2012).

### Duration of pharmacological treatment

There are no available data on the appropriate duration of treatment for an episode of PPP. The BAP consensus guidelines on using psychotropic medication in pregnancy and postpartum recommend following BAP or NICE guidelines for non-perinatal episodes of mania (McAllister-Williams et al., 2017). The BAP guideline for bipolar disorder recommends a minimum duration of 12 weeks for the short-term treatment of mania and recommends long-term treatment following a single severe episode of mania (Goodwin et al., 2016). NICE guidelines for bipolar disorder recommend reviewing the need for long-term treatment after 3–6 months of treatment for acute mania (National Institute for Health and Care Excellence (NICE), 2014b). The evidence-based UpToDate resource on the treatment of PPP recommends treating women for at least 1 year postpartum to reduce the risk of relapse, with a suggestion that some patients may require lifetime prophylaxis if there is risk of relapse without medication or in the presence of other risk factors such as suicidality (Payne, 2019).

### Psychosocial support

There are no studies examining the role of psychosocial interventions in PPP. In the absence of PPP-specific evidence, our recommendations have been extrapolated from the literature on mania and psychosis outside the perinatal period. Involving the partner/carer/support system with psychoeducation is an important aspect of PPP treatment, from the time of admission to the post-acute phase (Figure 1). An episode of PPP can lead to difficult emotions, role changes and relationship problems for the patient and partner. Individual and couples psychotherapy may help address these issues (Heron et al., 2012). There is evidence for the potential benefit of family-focused psychotherapy, cognitive behavioural therapy and interpersonal therapy in the maintenance phase of non-perinatal mania and psychosis, and these approaches can also be beneficial in PPP (Frawley et al., 2021; Goodwin et al., 2016; Miklowitz et al., 2021). Women recovering from PPP reported peer support to be an invaluable resource (Heron et al., 2012). National or local support groups for PPP are a useful resource for women and families (e.g. Action on Postpartum Psychosis, a UK national charity for families affected by PPP) (Action on Postpartum Psychosis, 2021).

Preconception counselling should be offered to women with a history of PPP or bipolar disorder who are planning a pregnancy. Improving awareness of PPP with inclusion in antenatal education classes can help women and families seek help earlier and prevent delays in accessing treatment (Action on Postpartum Psychosis, 2021).

Supporting the mother–infant dyad with bonding and development is an important aspect of post-acute care in PPP (Biaggi et al., 2021; Gilden et al., 2020b). Mother–infant interaction may be assessed during reviews with the mother–infant dyad. Where available, formal assessments of mother–infant interaction and parent–infant psychotherapy may be useful in PPP. With an aim to improve parental psychological symptoms and foster a stable parent–infant relationship, parent–infant psychotherapy can improve health outcomes for the mother–infant dyad (Barlow et al., 2016).

### Prognosis

A meta-analysis (of studies from 1949 to 1995) of long-term outcomes for women with PPP found that 40% had an isolated episode of PPP while 60% had a subsequent non-puerperal episode of mental illness during the follow-up period of 11–16 years (Gilden et al., 2020a). The majority of women (88%) in one study had returned to premorbid levels of functioning by nine months postpartum following PPP (Burgerhout et al., 2017). More recently, a prospective longitudinal study examined 106 women with first-onset mania or psychosis in the postpartum period (Rommel et al., 2021). In the 4-year follow-up period, 68% of women did not experience recurrence outside the postpartum period, with just 2% experiencing a recurrence exclusively following later pregnancies. Notably, the majority of women in this study were treated with lithium (76%) and antipsychotics (83%) during their MBU admission. In the same study, 32% of women experienced a relapse outside the postpartum period during the 4-year follow-up. Patients with a shorter duration of PPP have a more favourable prognosis, and a longer duration between index PPP and subsequent pregnancy predicts a further episode of PPP (Blackmore et al., 2013). A first episode of PPP also has a more favourable prognosis compared to a first episode of an affective disorder outside the perinatal period (Serretti et al., 2006).

## Conclusion

PPP is a psychiatric emergency necessitating rapid initiation of pharmacotherapy and admission. Although it is a discrete disorder on the bipolar spectrum with effects on both the mother and the infant, it is yet to be recognised as a distinct nosological entity by diagnostic manuals for psychiatric disorders. There is limited research into the aetiology and treatment of PPP. Current evidence for treatment in the acute phase points to antipsychotics, lithium and ECT. Lithium appears to have the strongest evidence for prophylaxis and relapse prevention. As prognosis is generally good when treatment is commenced promptly, further understanding of the risk factors and aetiology of PPP will enable early detection and timely management to reduce illness burden for the mother–infant dyad.
